# Structural insights into a high-fidelity CRISPR-Cas12a variant revealed using optimized graphene oxide cryo-EM grids

**DOI:** 10.1063/4.0001043

**Published:** 2025-10-27

**Authors:** Chhandosee Ganguly, Swarmistha Aribam, Leonard M. Thomas, Rakhi Rajan

**Affiliations:** Department of Chemistry and Biochemistry, Price Family Foundation Institute of Structural Biology, Stephenson Life Sciences Research Center, University of Oklahoma, Norman, Oklahoma, USA

## Abstract

CRISPR-Cas12a has become a widely used tool for genome editing and molecular diagnostics. However, unintended off-target DNA cleavage by Cas12a remains a key limitation. To address this, our lab engineered a Cas12a variant by modifying the bridge helix—a structurally important element—resulting in markedly reduced off-target activity.

To understand the structural basis of this enhanced specificity, we used cryo-electron microscopy (cryo-EM) to determine high-resolution structures of the variant. Initial grid preparation presented challenges in sample distribution and reproducibility. We overcame these by deriving a reproducible method for preparing graphene oxide (GO) coated Quantifoil holey carbon copper grids to achieve a sufficient area covered with the GO monolayer. These GO-coated grids significantly improved particle density within the holes while maintaining optimal ice thickness. This protocol may benefit other cryo-EM studies involving difficult samples. The resulting structures reveal novel conformational features of the bridge helix variant, offering mechanistic insights into how DNA cleavage specificity is modulated. These findings advance our understanding of Cas12a function and provide a structural framework for the rational design of next-generation genome editing tools with improved precision due to their reduced off-target effects.

